# Limitations of the Cough Sound-Based COVID-19 Diagnosis Artificial Intelligence Model and its Future Direction: Longitudinal Observation Study

**DOI:** 10.2196/51640

**Published:** 2024-02-06

**Authors:** Jina Kim, Yong Sung Choi, Young Joo Lee, Seung Geun Yeo, Kyung Won Kim, Min Seo Kim, Masoud Rahmati, Dong Keon Yon, Jinseok Lee

**Affiliations:** 1 Department of Biomedical Engineering, Kyung Hee University Seoul Republic of Korea; 2 Department of Radiology and Research Institute of Radiology, Asan Image Metrics, Clinical Trial Center, Asan Medical Center, University of Ulsan College of Medicine Seoul Republic of Korea; 3 Medical and Population Genetics and Cardiovascular Disease Initiative, Broad Institute of MIT and Harvard Cambridge, MA United States; 4 Department of Physical Education and Sport Sciences, Faculty of Literature and Human Sciences, Lorestan University Khoramabad Iran; 5 Department of Physical Education and Sport Sciences, Faculty of Literature and Humanities, Vali-E-Asr University of Rafsanjan Rafsanjan Iran; 6 Center for Digital Health, Medical Science Research Institute, Kyung Hee University Medical Center, Kyung Hee University College of Medicine Seoul Republic of Korea; 7 Department of Pediatrics, Kyung Hee University Medical Center, Kyung Hee University College of Medicine Seoul Republic of Korea; 8 Department of Electronics and Information Convergence Engineering, Kyung Hee University Yongin Republic of Korea

**Keywords:** COVID-19 variants, cough sound, artificial intelligence, diagnosis, human lifestyle, SARS-CoV-2, AI model, cough, sound-based, diagnosis, sounds app, development, COVID-19, AI

## Abstract

**Background:**

The outbreak of SARS-CoV-2 in 2019 has necessitated the rapid and accurate detection of COVID-19 to manage patients effectively and implement public health measures. Artificial intelligence (AI) models analyzing cough sounds have emerged as promising tools for large-scale screening and early identification of potential cases.

**Objective:**

This study aimed to investigate the efficacy of using cough sounds as a diagnostic tool for COVID-19, considering the unique acoustic features that differentiate positive and negative cases. We investigated whether an AI model trained on cough sound recordings from specific periods, especially the early stages of the COVID-19 pandemic, were applicable to the ongoing situation with persistent variants.

**Methods:**

We used cough sound recordings from 3 data sets (Cambridge, Coswara, and Virufy) representing different stages of the pandemic and variants. Our AI model was trained using the Cambridge data set with subsequent evaluation against all data sets. The performance was analyzed based on the area under the receiver operating curve (AUC) across different data measurement periods and COVID-19 variants.

**Results:**

The AI model demonstrated a high AUC when tested with the Cambridge data set, indicative of its initial effectiveness. However, the performance varied significantly with other data sets, particularly in detecting later variants such as Delta and Omicron, with a marked decline in AUC observed for the latter. These results highlight the challenges in maintaining the efficacy of AI models against the backdrop of an evolving virus.

**Conclusions:**

While AI models analyzing cough sounds offer a promising noninvasive and rapid screening method for COVID-19, their effectiveness is challenged by the emergence of new virus variants. Ongoing research and adaptations in AI methodologies are crucial to address these limitations. The adaptability of AI models to evolve with the virus underscores their potential as a foundational technology for not only the current pandemic but also future outbreaks, contributing to a more agile and resilient global health infrastructure.

## Introduction

In 2019, the outbreak of SARS-CoV-2 set off a global pandemic, substantially impacting human lifestyles and posing an unprecedented challenge to health care systems worldwide [1]. Given its high infectivity, the urgent need for rapid and accurate COVID-19 detection has become paramount for effective patient management, timely isolation, and implementation of appropriate public health measures. Consequently, numerous studies have been undertaken to devise efficient methods for detecting infection status quickly and easily. Among these studies, artificial intelligence (AI) models that leverage prominent symptoms, such as coughing, have shown potential [2,3]. The ability to discern COVID-19 solely based on analyzing recorded cough sounds presents a straightforward and swift approach to screening individuals. These AI models offer hope for rapid, large-scale screening and early identification of potential cases. However, it is essential to acknowledge certain limitations in these research trends, particularly regarding variations in symptoms attributed to different SARS-CoV-2 variants [4]. The emergence of new variants has introduced complexities in symptom profiles, making it more challenging to rely solely on cough sounds for accurate COVID-19 detection. As the virus continues to evolve, ongoing research and adaptations in AI models will be crucial to address these challenges and improve the accuracy and effectiveness of diagnostic approaches. Particularly, patients with the Omicron variant have shown a decreased occurrence of characteristic symptoms such as loss of taste and smell, while cold-like symptoms such as sneezing and a blocked nose have increased [5]. In essence, the symptoms of COVID-19 have been increasingly resembling those of a common cold. This implies that the previous AI models designed to detect COVID-19 using cough sounds may not work as effectively or may not be as reliable both currently and in the future.

## Methods

### Overview

In this study, we first used cough sounds as a diagnostic tool for COVID-19 stems from distinctive acoustic features that can differentiate between positive and negative cases. Building on prior research that used mel spectrograms to discern variance in cough sound spectra, our study adopts the variable frequency complex demodulation technique. Variable frequency complex demodulation offers a higher resolution than mel spectrograms, thus amplifying the discernibility of these patterns [6]. As depicted in Figure S1 in Multimedia Appendix 1, the cough sound spectra of patients with COVID-19 exhibit a unique irregular spectral intensity distribution, characterized by an initial decline followed by a subsequent rise over time. In contrast, the spectra from non–COVID-19 groups display a more uniform pattern, typically showing either a consistent distribution or a gradual decline in intensity. Furthermore, our analysis extends to the differentiation in audio signal features between the 2 patient groups. The examination of 5 selected audio signal features indicates clear distinctions, which are consistent across different data sets. Illustrated in Figure S2 in Multimedia Appendix 1 [6-9], we observe that the mean spectral roll-off for patients with SARS-CoV-2 positive trends toward lower values compared with that of negative individuals. Conversely, the mean spectral bandwidth is generally higher for those with COVID-19. Additional features, such as the SD of the spectral centroid, present lower values for positive cases, whereas the SD of spectral bandwidth is elevated, and the SD of the zero-crossing rate is reduced when compared with negative cases.

**Figure 1:**
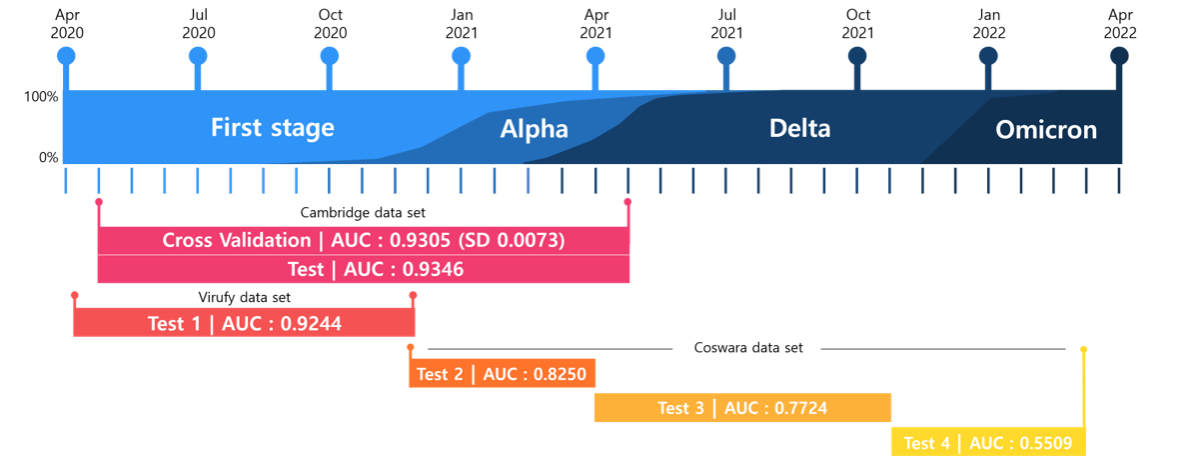
Area under the receiver operating curve (AUC) values for each data set and measurement period.

Furthermore, we aimed to demonstrate that an AI model trained on cough sound recordings from a specific period, especially the early stages of the COVID-19 pandemic, is not applicable to the ongoing situation with persistent variants. To illustrate these findings, we used 3 cough data sets named Cambridge, Coswara, and Virufy [5,10,11]. The Cambridge data set consists of audio recordings of variable-length cough audio collected through the COVID-19 Sounds App, developed by the University of Cambridge. The data measurement period encompasses the early stages of the wild type and Alpha variant occurrences (April 30, 2020, to April 26, 2021). We trained our AI model using the Cambridge data set. More specifically, we split the data into 8:2 for train and test data. Subsequently, for the train data, we performed 3-fold cross-validation and evaluated the model using the test data. The Virufy data set also includes crowdsourced cough sounds to identify patterns that signify respiratory diseases, such as COVID-19. The data measurement period primarily covers the early stages when the wild type was present (April 9, 2020, to November 26, 2020). We used the data set as additional test data. The Coswara data set also includes worldwide crowdsourced data collected through a website app, including cough, breath, and voice recordings for COVID-19 diagnosis. The data measurement period extended from the Alpha variant to the Omicron variant occurrences. We also used the data set as additional test data. To evaluate the performance of our AI model based on different variants, we categorized the data into 3 periods, each dominated by the Alpha, Delta, and Omicron variants, respectively. We summarized the data sets, along with the periods and COVID-19 variant status in which they were measured, in Figure 1 and Tables S1 and S2 in Multimedia Appendix 1 [6-9].

### Ethical Considerations

The protocol of this study was approved by the institutional review board of Kyung Hee University Hospital (KHUH 2022-06-042).

## Results

**Figure 2:**
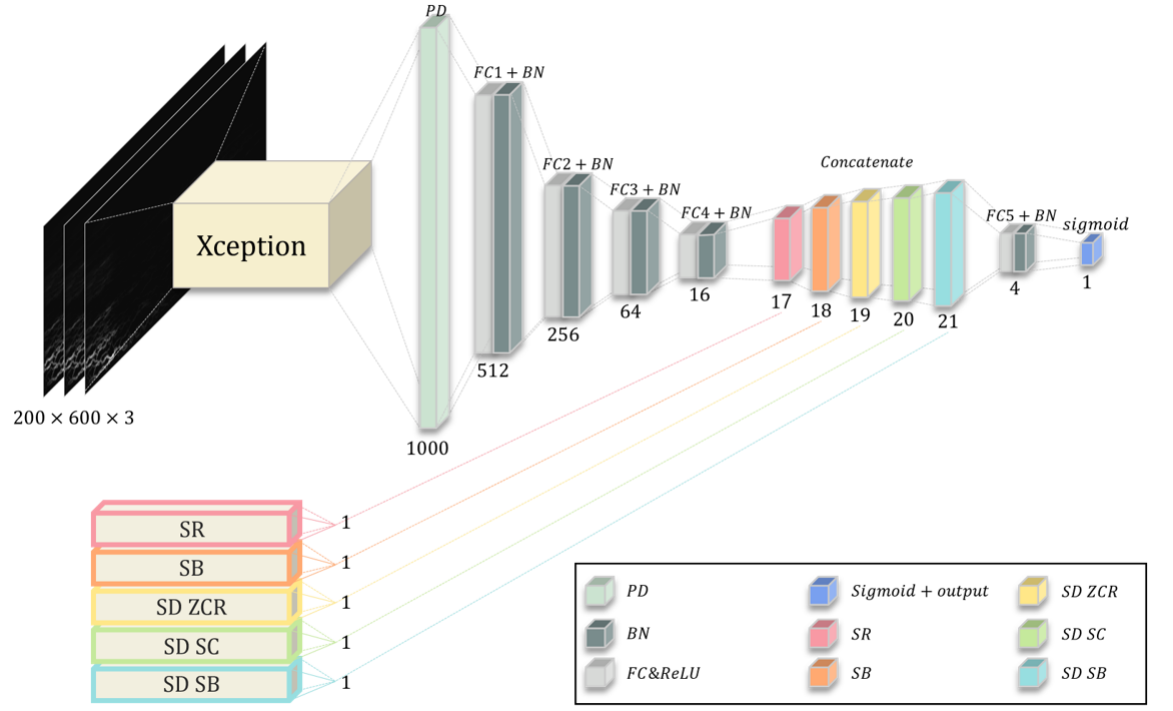
Our proposed model architecture. BN: batch normalization; FC: fully connected; PD: prediction; SB: spectral bandwidth; SD SC: SD of spectral centroid; SD ZCR: SD of zero crossing rate; SD SB: SD of spectral bandwidth; SR: spectral rolloff.

After the cough sound preprocessing, we developed an AI model to detect COVID-19 from extracted features and time-frequency spectrum using variable frequency complex demodulation (Multimedia Appendix 1 [6-9]). Table S2 in Multimedia Appendix 1 [6-9] summarizes the performance of our proposed AI model according to each data set and data measurement period. The results show that the cross-validation and test results from the Cambridge data set overlap, with an area under the receiver operating curve (AUC) of 0.93. Furthermore, the Virufy data set, which was measured around a similar period as the Cambridge data set, also demonstrated a similar performance: the AUC was 0.92. On the other hand, in the case of the Coswara data set, divided into 3 groups based on the variants, it was observed that the AUC for Alpha was 0.83; for Delta, 0.77; and for Omicron, 0.55; this indicates a decline in performance as the variants progressed. Figures 1 and 2 summarize the overall performance for each data set and measurement period.

## Discussion

The fight against COVID-19 has been marked by the deployment of various traditional diagnostic methods, each serving as a critical pillar in our response to the pandemic. Central among these is the reverse transcription-polymerase chain reaction test, widely regarded as the gold standard for its high specificity and sensitivity [12,13]. Rapid antigen tests and serological assays have also been instrumental, offering quick screening and insight into past infections, respectively. Despite their strengths, these methods have limitations, including resource dependency, time constraints, and varying degrees of accuracy. In this context, AI has emerged as a powerful aid, augmenting traditional diagnostic methods and addressing their shortcomings. By facilitating enhanced data analysis and interpretation, AI algorithms have the potential to streamline reverse transcription-polymerase chain reaction workflows, improve the reliability of antigen tests, and provide a more nuanced understanding of serological data. The integration of AI extends further to the analysis of medical imaging, where it aids in identifying patterns indicative of COVID-19, thus offering a valuable complement to molecular testing [14-16].

Our research has focused on the novel application of AI in analyzing cough sounds, a symptom-based method with promise for noninvasive, rapid screening. We demonstrated that AI models, when trained on cough sounds, could provide a swift approach to preliminary screening, though their performance varied with the emergence of new variants. Despite the performance variability, the inherent adaptability of AI models ensures their lasting relevance. These models can be reconfigured as pretrained frameworks ready to be fine-tuned against emerging viral strains. Such adaptability allows for the rapid deployment of AI tools in response to evolving pathogens, showcasing the potential of AI to serve not only the current pandemic but also as a foundational technology for future outbreaks. The capacity of these AI models to evolve in step with the virus paves the way for an agile diagnostic ecosystem that can quickly adapt to new threats, ultimately contributing to a more resilient global health infrastructure.

## Appendix

Multimedia Appendix 1 Supplemental tables and figures.

## Abbreviations

AI: artificial intelligence
AUC: area under the receiver operating curve

## Data Availability

The data sets generated or analyzed during this study are available from the corresponding author on reasonable request.
