# Supplementary material for: Limitations of the Cough Sound-Based COVID-19 Diagnosis Artificial Intelligence Model and its Future Direction: Longitudinal Observation Study
Source: J Med Internet Res. 2024 Feb 6;26:e51640. doi: 10.2196/51640 (PMC10879967; doi:10.2196/51640)
Supplement: Multimedia Appendix 1 [file jmir_v26i1e51640_app1.pdf]

Supplementary Materials

Preprocessing

The raw data contained multiple instances of cough sounds. Therefore, cough detection was performed to segment them into individual instances. Subsequently, the signals were resampled at a frequency of 44.1 kHz. Each raw cough data $C_i(t)$ is normalized using Equation (1) and (2),

$$C_i(t) = C_i(t) - mean(C_i(t)) \tag{1}$$

$$C_i(t) = 2 \times \frac{C_i(t) - \min(C_i(t))}{\max(C_i(t)) - \min(C_i(t))} - 1 \tag{2}$$

where i represents the number of cough data.

For the input image of our deep learning model, we extracted the time-frequency spectrum from the cough signals using the variable frequency complex demodulation (VFCDM) algorithm[1]. VFCDM image enables a high-resolution time-frequency spectrum image that enables the observation of power variations of different frequencies over time with high resolution. In our study, we used a VFCDM image with dimensions of 200x600 and converted it into grayscale, with a range of (0,1).

To include more information about cough sounds in the input data, we extracted features such as spectral roll-off (SR), spectral bandwidth (SB), zero crossing rate (ZCR), spectral centroid (SC). Spectral roll-off refers to the frequency at which a certain proportion of the energy spectrum is located. In our study, we used a threshold of 0.85 as the reference ratio. Spectral centroid represents the frequency value that serves as the center of the energy spectrum. Spectral bandwidth indicates the range of frequencies where the energy of the spectrum reaches half of its maximum intensity (Multimedia Appendix 1, Figure S3). Zero crossing rate is the rate at which the amplitude sign of a signal changes in the time domain. These features are

commonly used in audio signal analysis[2]. Cough can be divided into three phases: inspiratory, compressive, and expiratory[3]. Therefore, based on these phases, we divided the frames required for each feature extraction into three equal parts. We calculated the average and standard deviation of these three frame values and used them as the final feature data.

AI Model to Detect COVID-19

Figure 2 summarizes the architecture of our proposed AI model for the early diagnosis of COVID-19 based on cough sounds. We used Xception as a pre-trained model with three identical VFCDM images as the input. Xception has 14 modules with a total of 36 convolution layers and linear residual connections to classify images into 1000 object categories[4]. We used the convolutional layers followed by a sequence of fully connected layers (FC) with 512, 256, 64, and 16 nodes, respectively. We used a rectified linear unit (ReLU) as an activation function. Subsequently, we concatenated the frame mean value of SR and SB, along with the frame standard deviation value of ZCR, SC, and SB. We then performed binary classification by adding an additional FC layer and applying the sigmoid function.

Performance Evaluation

For performance evaluation, accuracy metrics such as sensitivity, specificity, F1 score, precision, and balanced accuracy are used. These are defined as follows:

$$Sensitivity = \frac{TP}{TP\ + FN} \tag{3}$$

$$Specificity = \frac{TN}{TN\ + FP} \tag{4}$$

$$\text{F1 score} = 2 \cdot \frac{precision \cdot recall}{precision + recall} \tag{5}$$

$$\text{Precision} = \frac{TP}{TP + FP} \tag{6}$$

$$Balanced\ Accuracy = \frac{Sensitivity + Specificity}{2} \tag{7}$$

TP, TN, FP, and FN represent the true positive, true negative, false positive, and false negative. Additionally, we used area under the receiver operation characteristic (AUROC).

Supplementary reference

1. Hengliang W, Kin S, Kihwan J, Chon KH. A High Resolution Approach to Estimating Time-Frequency Spectra and Their Amplitudes. Annals of biomedical engineering. 2006;34. doi: 10.1007/s10439-005-9035-y.
2. G S, K U, S K. Trends in audio signal feature extraction methods. Applied Acoustics. 2020;158. doi: 10.1016/j.apacoust.2019.107020.
3. B CA. The physiology of cough. Paediatric respiratory reviews. 2006;7:2–8. doi: https://doi.org/10.1016/j.prrv.2005.11.009.
4. Chollet F. Xception: Deep Learning with Depthwise Separable Convolutions. 2017 IEEE Conference on Computer Vision and Pattern Recognition (CVPR): IEEE Computer Society; 2017. p. 1800-7.

Figure S1. VFCDM image between positive and negative patients

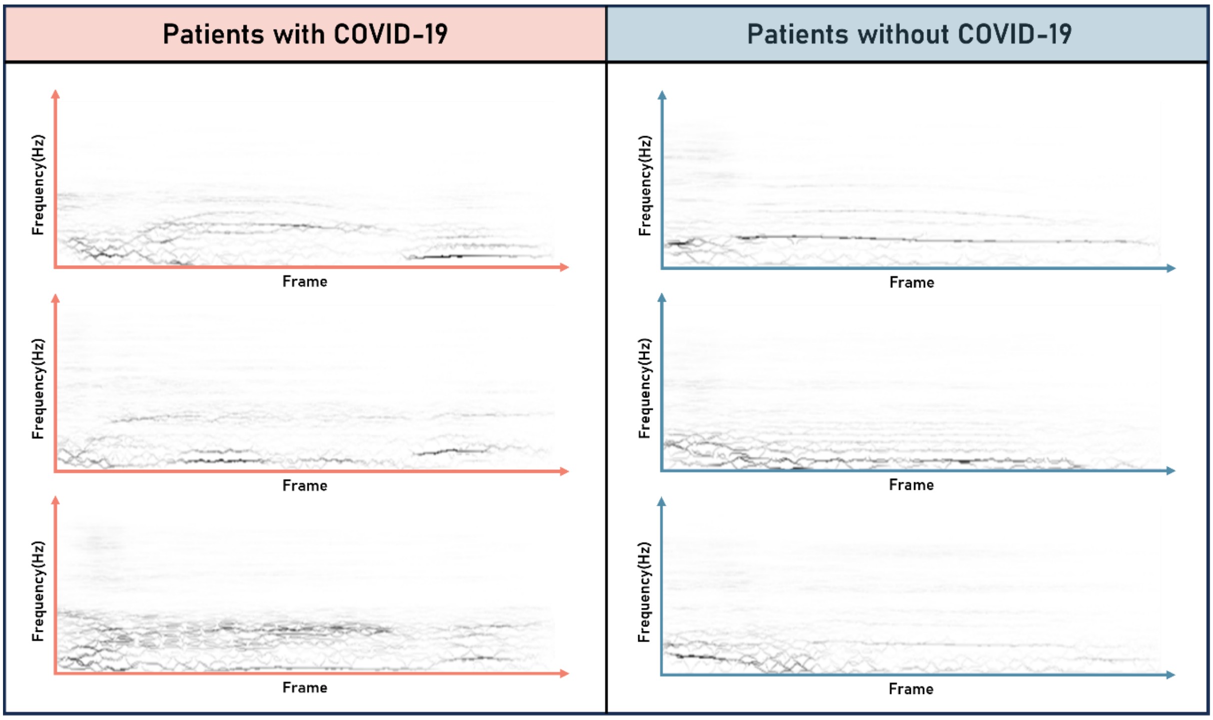

Figure S2. Cough audio signal feature between positive and negative patients in Cambrige and Virufy dataset.

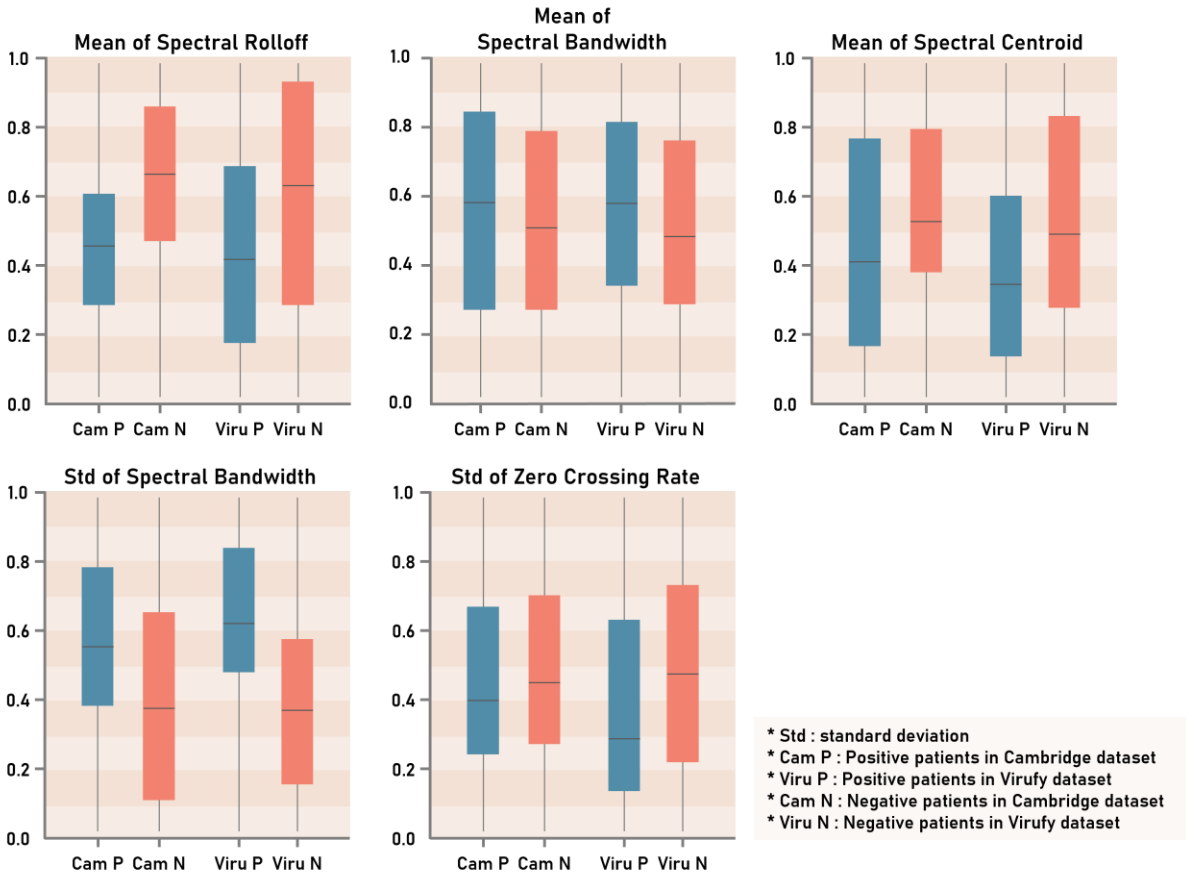

Figure S3. Visualization of spectral features.

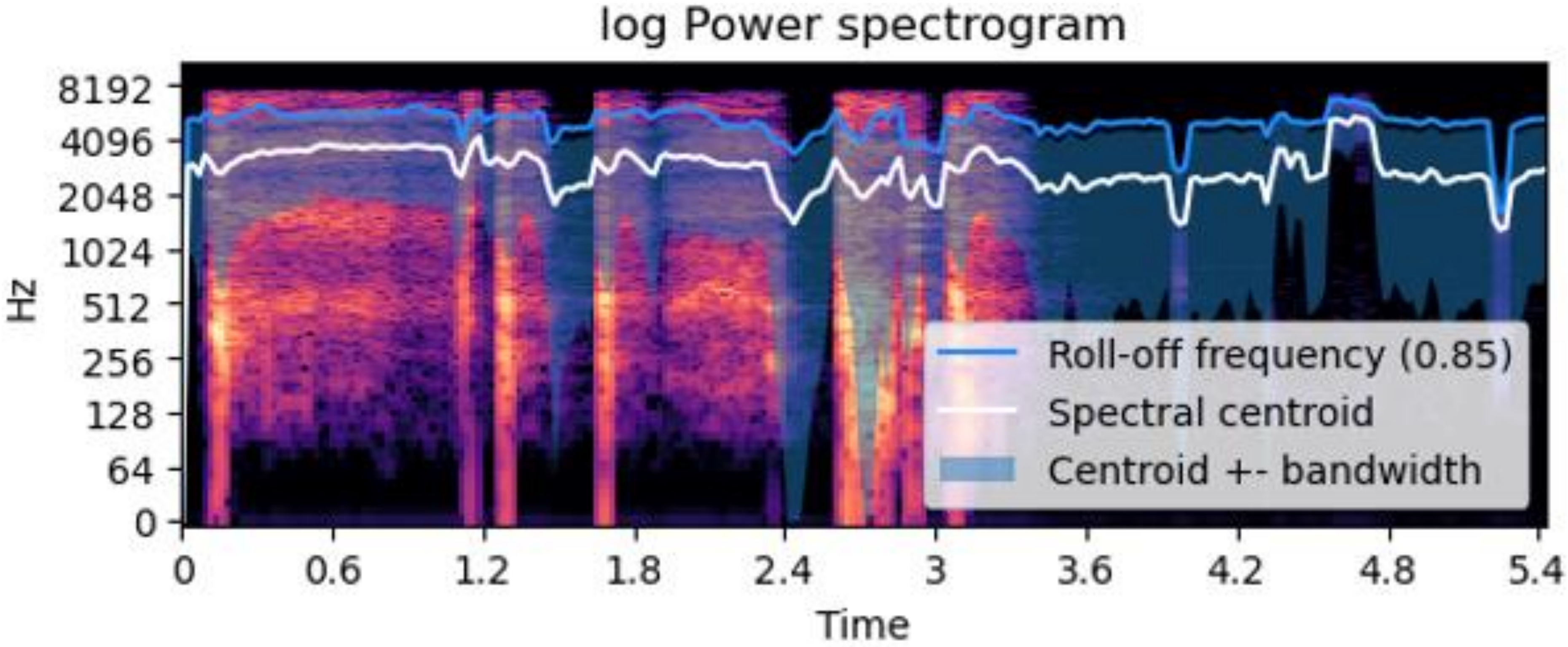

Table S1. Three datasets along with the periods and COVID-19 variant status.

Dataset		Subjects	Period	Variation	Train/Test
**Cambridge**	COVID-19	75	2020.04.30 to 2021.04.26	First stage & Alpha	Training with 3-fold cross validation (80%)<br>Test set (20%)
	Non-COVID-19	388			
**Virufy**	COVID-19	1540	2020.04.09 to 2020.11.26	First stage	Test set
	Non-COVID-19	124			
**Coswara**	COVID-19	5	2020.10.31 to 2021.02.06	Alpha	Test set
	Non-COVID-19	8			
	COVID-19	109	2021.04.06 to 2021.08.30	Delta	Test set
	Non-COVID-19	21			
	COVID-19	167	2021.09.14 to 2022.02.24	Omicron	Test set
	Non-COVID-19	21			

Table S2. Cross validation results and test data results according to each dataset and measurement period.

Dataset	Variant	CV/Test	Sensitivity	Specificity	F1 score	Precision	Balanced Accuracy	AUROC
**Cambridge**	First stage & Alpha	CV	0.9333 ± 0.0000	0.9487 ± 0.1282	0.8490 ± 0.0257	0.7794 ± 0.0434	0.9410 ± 0.0064	0.9305 ± 0.0073
		Test	0.9333	0.9231	0.8000	0.7000	0.9247	0.9346
**Virufy**	First stage	Test	0.8065	0.9883	0.8264	0.8475	0.8974	0.9244
**Coswara**	Alpha	Test	1.0000	0.7500	0.8333	0.7143	0.8750	0.8250
	Delta	Test	0.6239	0.8571	0.7556	0.9577	0.7405	0.7724
	Omicron	Test	0.2156	0.8095	0.3478	0.9000	0.5125	0.5509
